# What the papers say

**DOI:** 10.1093/jhps/hnw028

**Published:** 2016-08-17

**Authors:** Ajay Malviya

**Affiliations:** Northumbria Healthcare NHS Foundation Trust, Woodhorn Lane, Ashington, NE63 9JJ

## INTRODUCTION

The *Journal of Hip Preservation Surgery* (*JHPS*) is not the only place where work in the field of hip preservation may be published. Although our aim is to offer the best of the best, we continue to be fascinated by work that finds its way into journals other than our own. There is much to learn from it so *JHPS* has selected six recent and topical articles for those who seek a brief summary of what is taking place in our ever-fascinating world of hip preservation. What you see here are the mildly edited abstracts of the original articles, to give them what *JHPS* hopes is a more readable feel. If you are pushed for time, what follows should take you no more than 10 min to read. So here goes …

## DOES HIP ARTHROSCOPIC INTERVENTION MEET PATIENT EXPECTATIONS?

Multi-centred research in the United States [1] has looked at hip arthroscopy outcomes with respect to patient acceptable symptomatic state (PASS) and minimal clinically important difference (MCID). The authors performed a systematic review of studies that had used the modified Harris Hip Score (mHHS) and/or Hip Outcome Score (HOS) outcomes with at least 1-year of follow-up. Ninety-one studies (9746 hips) were included for review. Eighty-one studies (9317 hips) contained only primary hip arthroscopies and were the focus of this review. The remaining studies (429 hips) did not exclude patients with prior surgical history and were thus considered separately. Mean mHHS, HOS-ADL (Activities of Daily Living) and HOS-SS (Sports-Specific) scores were compared with previously published PASS and MCID values.

After 31(± 20) months, 5.8% of study populations required revision arthroscopy and 5.5% total hip arthroplasty (THA). A total of 88, 25 and 30% of study populations met PASS for mHHS, HOS-ADL, and HOS-SS, respectively, and 97, 90 and 93% met MCID. On bivariate analysis, increasing age was associated with significantly worse post-operative mHHS (*P* < 0.01, *R*^2 ^=0.14), HOS-SS (*P* = 0 0.05, *R*^2^= 0.12), and rates of reoperation (*P* = 0.02, *R*^2^= 0.08). Increasing body mass index was associated with significantly worse HOS-ADL (*P* = 0.02, *R*^2^= 0.35) and HOS-SS (*P* = 0.03, *R*^2 ^=^ ^0.30).

The authors concluded that after a meta-analysis of 81 studies of primary hip arthroscopy, they have found that >90% of study populations meet MCID standards for the most commonly used patient-reported outcomes measures in hip arthroscopy literature, mHHS and HOS. Eighty-eight percent meet PASS standards for the mHHS, but PASS standards are far more difficult to achieve for HOS-ADL (25%) and HOS-SS (30%) subscales. The authors postulated that differences in psychometric properties of the mHHS and HOS likely account for the discrepancies in PASS. It maybe that although pain and function improve after surgery it does not meet patient expectations in terms of their activity of daily living or engagement in sporting activities.

## OSTEOARTHRITIC CHANGES RATHER THAN AGE PREDICT OUTCOME FOLLOWING ARTHROSCOPIC TREATMENT OF FEMOROACETABULAR IMPINGEMENT IN MIDDLE-AGED PATIENTS

Herrman *et al.* [2] from Freiburg, Germany evaluated the outcome following arthroscopic treatment of femoroacetabular impingement (FAI) in middle-aged patients and defined risk factors for conversion to THA.

This was a retrospective case series of 79 consecutive patients (40–65 years) undergoing arthroscopic treatment of FAI at a minimum follow-up of 12.

Outcome was assessed using HOS. Alpha angle, Kellgren Lawrence grade (K-L grade), joint space ( JS) width, lateral centre edge (LCE) angle, caput-collum-diaphysis (CCD) angle and acetabular index (AI) were analysed retrospectively. THA group and Non-THA group were compared.

Seventy-nine patients (mean age 48.6 years, mean follow-up 32 months) were included. Eighteen patients (22.8%) were converted to THA. Mean HOS score in the Non-THA group at time point of follow-up was 80.2. Non-THA group and THA group showed no significant differences for mean age (48.2 years versus 49.9 years, *P* = 0.278), alpha angle (*P* = 0.541), LCE (*P* = 0.294), CCD (*P* = 0.101) and AI (*P* = 0.661) in contrast to differences for JS (*P* ≤ 0.001) and K-L grade (*P* ≤ 0.001). Risk of conversion to THA was higher for patients with K-L grade 3 (*P* = 0.003) or JS ≤2 mm (*P* = 0.001).

The authors concluded that one fifth of the middle-aged patients required early conversion to THA. Advanced JS narrowing and grade of osteoarthritis rather than age alone can be considered as risk factor for conversion to THA.

## WOULD PSYCHOLOGICAL COUNSELING IMPROVE PATIENT OUTCOME AFTER HIP PRESERVATION SURGERY?

Richard *et al.* [3] from Texas Scottish Rite Hospital, Dallas, United States looked at the role of perioperative interdisciplinary intervention to modify outcome of adolescents undergoing hip preservation surgery (HPS). Adolescent HPS candidates typically present with chronic pain compounded by a high rate of psychological symptoms and maladaptive behaviour, which can negatively affect psychological function and surgical outcomes. In this study, they have quantified psychological and functional improvements in these patients from pre-operative presentation to post-operative follow-up by using an integrated interdisciplinary approach.

A total of 67 patients undergoing HPS were evaluated pre-operatively and post-operatively at one year by staff psychologists. Perioperative psychological intervention consisted of education, counseling, and administration of self-report measures. Self-report measure scores were compared pre-operatively and post-operatively, grouped by orthopaedic diagnoses. Frequency analysis, correlational analysis, and analysis of variance were conducted.

The study found that psychological function improved significantly at follow-up: decreased emotional symptomatology (46.1–43.6, *P* = 0.013), anxiety (49.6–45.8, *P* < 0.001), school problems (46.6–44.7, *P* = 0.035), internalizing problems (46.3–44.1, *P* = 0.015), social stress (44.5–42.3, *P* = 0.024), sense of inadequacy (49.0–46.0, *P* = 0.004), and increased self-concept (51.1–54.1, *P* = 0.003). Resiliency factors also significantly improved: increased mastery (50.3–52.9, *P* = 0.001) and resourcefulness (49.7–52.0, *P* = 0.046), decreased emotional reactivity (46.3–42.9, *P* = 0.001), and vulnerability (47.7–44.7, *P* = 0.011). Physical function and return to activity also significantly improved (University of California-Los Angeles: 7.1–8.7, *P* = 0.017; mHHS: 67.3–83.8, *P* < 0.001). Return to activity positively correlated with optimism and self-efficacy (*P* = 0.041). FAI and hip dysplasia patients consistently reported feeling less depressed (*P* = 0.036), having fewer somatic complaints (*P* = 0.023), fewer internalized problems (*P* = 0.037), and exhibiting fewer atypical behaviours (*P* = 0.036) at follow-up. Slipped capital femoral epiphysis patients did not demonstrate improvements in psychological functioning post-operatively.

The authors concluded that perioperative psychological education and counseling, in combination with HPS, improved post-operative psychological and physical function. Patients reported reduced anxiety, school problems, and social stress, with marked increase in resilience. Increased mobility and return to activity significantly correlated with improved optimism and self-efficacy. The study highlights a problem that we see in the older population as well and it may set a background for a catastrophization study for this complex group of patients.

## WHAT DOES THE LITERATURE CONCLUDE ABOUT HIP ARTHROSCOPY IN THE SETTING OF DYSPLASIA?

Hip arthroscopy in the setting of hip dysplasia is controversial in the orthopaedic community, as the outcome literature has been variable and inconclusive. Researchers [4] from the McMaster’s University, Hamilton, Canada have performed a systematic review with the hypothesis that outcomes of hip arthroscopy may be diminished in the setting of hip dysplasia, but outcomes may be acceptable in milder or borderline cases of hip dysplasia.

A systematic search was performed in duplicate for studies investigating the outcome of hip arthroscopy in the setting of hip dysplasia up to July 2015.

Study parameters including sample size, definition of dysplasia, outcomes measures, and re-operation rates were obtained. Information was collected on the levels of evidence and furthermore a quality assessment was performed.

The systematic review identified 18 studies investigating hip arthroscopy in the setting of hip dysplasia, with 889 included patients. Criteria used by the studies to diagnose hip dysplasia and borderline hip dysplasia included centre edge angle in 72% of studies but the range of angles were quite variable. Although 89% of studies reported improved post-operative outcome scores in the setting of hip dysplasia, revision rates were considerable (14.1%), with 9.6% requiring conversion to THA.

The available orthopaedic literature suggests that although improved outcomes are seen in hip arthroscopy in the setting of hip dysplasia, there is a high rate of re-operation and conversion to THA. Furthermore, the criteria used to define hip dysplasia vary considerably among published studies and the orthopaedic literature needs clarification on this aspect. One also needs to be mindful that the published literature would typically be from a big centre, in experienced hands and may not have the similar results in less experienced surgeons.

## DO LABRAL TEARS INFLUENCE POOR OUTCOMES AFTER PERIACETABULAR OSTEOTOMY FOR ACETABULAR DYSPLASIA?

Hagio *et al.* [5] from Fukuoka, Japan note that acetabular dysplasia is frequently associated with intra-articular pathology such as labral tears (LTs), but whether LTs should be treated at the time of periacetabular osteotomy (PAO) remains controversial. The purpose of this study was to compare the clinical outcomes and radiographic corrections of PAO for acetabular dysplasia between patients with and without LTs pre-operatively.

This retrospective study includes 70 hips in 67 patients with acetabular dysplasia who underwent PAO. Of 47 hips (45 patients) with LTs pre-operatively, 27 (25 patients) underwent PAO alone, and were classified as the LT alone group, and 20 (20 patients) underwent combined PAO and osteochondroplasty, and were classified as the labral tear osteochondroplasty (LTO) group. The non-LT group included 23 hips in 22 patients.

The study reports that there were no significant differences between groups for post-operative Harris hip scores, degree of progression of osteoarthritis or rate of reoperation. The pre-operative alpha angle was significantly larger in the LTO group compared with the other groups (*P* < 0.0001).

The authors concluded that PAO provides equivalent short-term relief of pain and functional outcome in patients with or without LTs. The rate of progression of osteoarthritis and reoperation was not significantly increased in patients with LTs. With the use of minimally invasive approach intra-articular hip pathology is typically left undisturbed at the time of PAO and this study backs this philosophy.

## EFFECTS OF SULFUR BATH ON HIP OSTEOARTHRITIS: A RANDOMIZED, CONTROLLED, SINGLE-BLIND, FOLLOW-UP TRIAL

Kovacs *et al.* [6] from Budapest, Hungary, have evaluated the effects of balneotherapy in patients with osteoarthritis of the hip. This randomized, controlled, investigator-blinded study enrolled outpatients with hip osteoarthritis according to American College of Rheumatology (ACR) criteria. In addition to home exercise therapy, one patient group received balneotherapy for 3 weeks on 15 occasions. The mineral water used in this study is one of the mineral waters with the highest sulfide ion content (13.2 mg/l) in Hungary. The control group received exercise therapy alone. The WOMAC Likert 3.1 index and the EQ-5D quality of life self-administered questionnaire were completed three times during the study: prior to first treatment, at the end of the 3-week treatment course, and 12 weeks later. The main endpoint was achievement of Minimal Clinically Important Improvement (MCII) at 12 weeks, defined as ≥7.9 points in a normalized WOMAC function score. The intention to treat analysis included 20 controls and 21 balneotherapy patients. At 12 weeks, 17 (81%) balneotherapy group patients had MCII and 6 (30%) of controls (*P* = 0.001). Comparing the results of the two groups at the end of treatment, there was a significant difference in the WOMAC stiffness score only, whereas after 12 weeks, the WOMAC pain, stiffness, function, and total scores also showed a significant difference in favor of the balneotherapy group. The difference between the two groups was significant after 12 weeks in point of EQVAS score, too. The results of our study suggest that the combination of balneotherapy and exercise therapy achieves more sustained improvement of joint function and decreases in pain than exercise therapy alone.

It may be that surgery or physiotherapy is not the solutions one should be looking for!
